# An Effective Method to Accurately Extract the Parameters of Single Diode Model of Solar Cells

**DOI:** 10.3390/nano11102615

**Published:** 2021-10-04

**Authors:** Zhaoxu Song, Kun Fang, Xiaofang Sun, Ying Liang, Wei Lin, Chuanzhong Xu, Gongyi Huang, Fei Yu

**Affiliations:** College of Information Science and Engineering, Huaqiao University, Xiamen 361021, China; hquszx@163.com (Z.S.); fksaya@126.com (K.F.); xfsun@hqu.edu.cn (X.S.); liangyinghqu@163.com (Y.L.); linwei_0311@126.com (W.L.); xucz@hqu.edu.cn (C.X.); hgy@hqu.edu.cn (G.H.)

**Keywords:** solar cells, parameter extraction, single diode model, non-iterative

## Abstract

A non-iterative method is presented to accurately extract the five parameters of single diode model of solar cells in this paper. This method overcomes the problems of complexity and accuracy by simplifying the calculation process. Key parts of the equation are to be adjusted dynamically so that the desired five parameters can be obtained from the *I-V* curve. Then, the *I-V* and *P-V* characteristic curves of solar cells are used to compare the effectiveness of this method with other methods. Furthermore, the root mean square error analysis shows that this method is more applicable than other methods. Finally, the *I-V* and *P-V* characteristics simulated by using the extracted parameters in this method are compared and discussed with the experimental data of solar cells under different conditions. In fact, this extraction process can be regarded as an effective and accurate method to estimate solar cells’ single diode model parameters.

## 1. Introduction

With the intensification of the greenhouse effect, the demands for clean and sustainable energy resources are sharply increasing worldwide and this has become a public concern [1]. Solar energy is undoubtedly one of the most promising, pollution-free energy sources. Because of solar cells’ advantages of energy saving and no pollution, the single diode model of solar cells has become one of the hottest research projects. The main purpose of these studies is to ascertain an analytical solution [2] and parameter extraction [3] to predict the *I-V* and *P-V* characteristics of solar cells. At present, analytical solution algorithms have been developed for many years, and the technology tends to be mature and saturated. However, parameter extraction routines still have to face a challenge for a trade-off between accuracy and efficiency. In fact, the accuracy of the single diode model predictions for solar cells’ characteristics are fully dependent on the model parameter values being extracted. Although complex extraction [4] procedures can obtain high precision parameter values, it may lead to inefficiency of computational process. Therefore, the single diode model of solar cells urgently needs an accurate and effective method to extract the model parameters.

Up until now, several authors have proposed various methods to determine different parameters in the single diode model. These methods can be divided into two categories [5,6]. One category is non-iterative analysis procedures [7,8,9,10,11,12,13,14,15,16,17,18,19,20,21,22], which are reviewed in [23]. They determine the analytical solution by simplifying and replacing the key parts of the equation, and then calculating the parameter values by depending on the information in the datasheet provided by the manufacturer [24,25,26], which refers to short circuit current, open circuit voltage, maximum power point, or the slope of the intersection of the *I-V* characteristic curve and the coordinate axes. Although these approaches are relatively simple and the calculation process is fast, the simplification and replacement often lead to a lack of accuracy and to results without physical significance [27]. Additionally, since the parameters are only obtained from the data in the datasheet, the results obtained are also very sensitive to the measurement error. These measurement errors are caused by the different accuracy of the test equipment. Different significant figures will also have a certain impact on the accuracy of the results. The other category is numerical or intelligent algorithm programs [28,29,30,31,32]. These are essentially processes of optimization or fitting, which can minimize the error between the obtained *I-V* or *P-V* characteristic curves and the experimental data, and then obtain high-precision parameter values. However, the inefficiency of the calculation process has always been the biggest problem for this kind of extraction strategy. Briefly, all these methods are almost difficult to have a good trade-off between accuracy and efficiency. Therefore, an efficient and accurate parameter extraction program is still needed to embed the circuit simulator of the model and diagnose the process optimization problem.

In this paper, an effective non-iterative method is proposed to accurately extract five parameters in solar cells’ single diode model. The analytical solution of the terminal current-voltage equation of the equivalent circuit model is firstly derived. Subsequently, five basic parameter equations are listed according to data obtained from *I-V* curve. Then, the important parts of the basic circuit equations are simplified and replaced to obtain the five expressions of the parameters. Finally, the five extracted parameters are substituted into the analytical solution to simulate the *I-V* and *P-V* characteristics of solar cells. Simultaneously, five parameter extraction methods described in other works of the literature are compared with the method proposed in this paper. The obtained parameter values and the RMSE are recorded. Furthermore, a comprehensive experimental evaluation is conducted to demonstrate the accuracy and verify the effectiveness of the proposed approach based on different solar cells’ photovoltaic technologies, irradiances, and temperatures. The results show that this accurate and efficient strategy can play a good role in single diode model parameter extraction. In fact, the method proposed in this paper is easier to be used to be implement lumped parameter model into simulators in technology. In addition, it also helps to provide an optimization suggestion on solar cells’ preparations.

## 2. Method of Parameter Extraction

**Figure 1:**
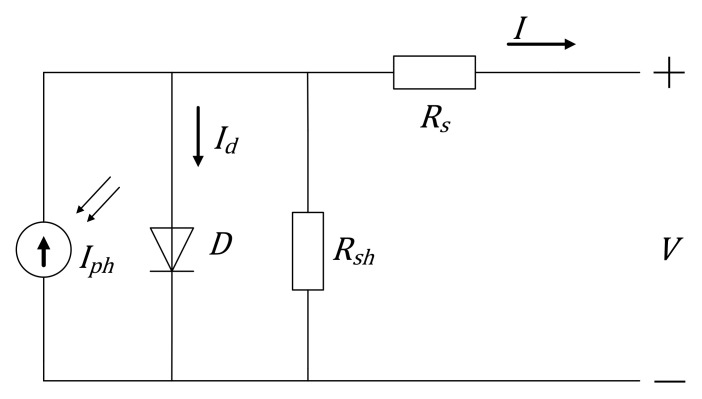
Single diode equivalent circuit model of solar cells.

The equivalent circuit model of the single diode of solar cells is shown in Figure 1, including a photocurrent source, a single diode, a series resistance, and a shunt resistance. The five parameters of the model are the photocurrent *I_ph_*, the diode reverse saturation current *I_s_*, the diode ideal factor *n*, the parallel resistance *R_sh_*, and the series resistance *R_s_*. According to Kirchhoff’s current law and Schockley’s ideal diode current equation, the terminal current-voltage equation of the circuit model is deduced as follows:

(1) $$I=Iph-\left(\frac{V+IRs}{Rsh}\right)-Is\left(\exp\left(\frac{V+IRs}{nVT}\right)-1\right)$$

In Equation (1), *V_T_* is the thermal voltage, which can be calculated by *V_T_* = *kT*/*q*, where *k* is the Boltzmann constant, *T* is the cell temperature, and *q* is the charge of the electron. According to Equation (1), the main purpose of this research is to adjust these five parameters *I_ph_*, *R_s_*, *R_sh_*, *I_s_*, *n* to predict the *I-V* characteristics, so that they are consistent with the electrostatic performances of solar cells. These adjustments are usually based on the data on the *I-V* curves measured by experiments or on the datasheet provided by the manufacturer.

**Figure 2:**
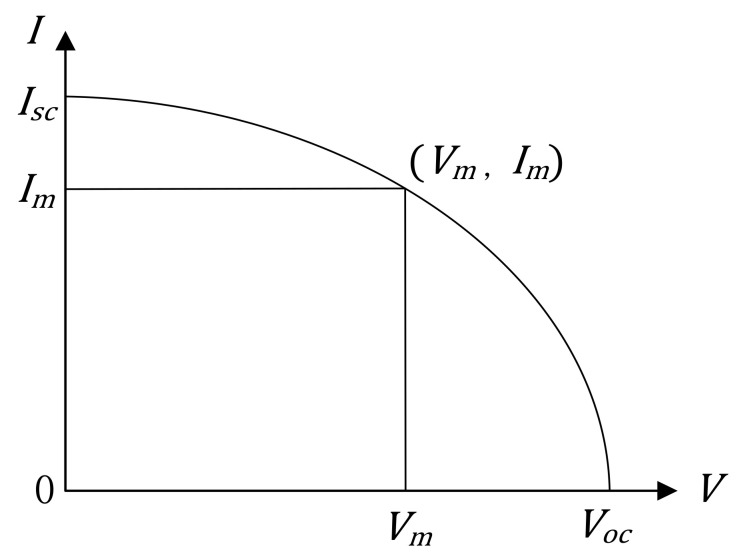
*I-V* curve of single diode model in solar cells.

The typical *I-V* curve of solar cells’ single diode model is shown in Figure 2. There are three important points, i.e., the short-circuit point, the open-circuit point, and the maximum power point. In fact, the voltage (*V*) and current (*I*) values of the three points are the basic template data and always known from solar cells’ data sheet, so they are hence used to create relevant equations as follows.

At the short-circuit point: (*V* = 0, *I* = *I_sc_*), Equation (1) can be represented as

(2) $$Iph=Isc+Is\left(\exp\left(\frac{IscRs}{nVT}\right)-1\right)+\frac{IscRs}{Rsh}$$

At the open-circuit point: (*V* = *V_oc_*, *I* = 0), Equation (1) can be written by

(3) $$Iph=\frac{Voc}{Rsh}+Is\left(\exp\left(\frac{Voc}{nVT}\right)-1\right)$$

At the maximum power point: (*V* = *V_m_*, *I* = *I_m_*), these values are substituted into Equation (1), yielding:

(4) $$Iph=\frac{Vm+ImRs}{Rsh}+Im+Is\left(\exp\left(\frac{Vm+ImRs}{nVT}\right)-1\right)$$

Under the same irradiance, the left side of Equations (3) and (4) is the same, which means that their right side is equal, yielding:

(5) $$Is\exp\left(\frac{Voc}{nVT}\right)+\frac{Voc-Vm}{Rsh}-Im-\frac{RsIm}{Rsh}-Is\exp\left(\frac{Vm+RsIm}{nVT}\right)=0$$

Generally, these three equations, i.e., Equations (2), (3), and (5), are not enough for extracting the five parameters of the model. Thus, two supplementary equations have to be added to establish an equation set consisting of five equations. In Figure 1, the shunt and series resistances *R_sh_* and *R_s_* are estimated as the experimental resistances *R_sho_* and *R_so_*, which are usually calculated from the slope of the *I-V* curve at short circuit (SC) and open circuit (OC). Therefore, two supplementary equations are written as

(6) $$Rsho=-{\left.\frac{dV}{dI}\right|}_{SC}$$

(7) $$Rso=-{\left.\frac{dV}{dI}\right|}_{OC}$$

Here, *R_sho_* and *R_so_* can be easily approximated by

(8) $$Rsho\approx -\frac{0.001}{f\left(0.001\right)-Isc}\left(\Omega \right)$$

(9) $$Rso\approx -\frac{0.001}{0-f\left(Voc-0.001\right)}\left(\Omega \right)$$

It is noted that *f* is the function of the *I-V* curve in Figure 2. Then, by deriving Equation (1) at the short-circuit point to obtain the expression of ${\left.\frac{dV}{dI}\right|}_{SC}$ and substituting it into Equation (6), Equation (10) can be obtained as

(10) $$\frac{Rsho-Rs}{Rsh}+\frac{Is}{nVT}\left(Rsho-Rs\right)\exp\left(\frac{IscRs}{nVT}\right)-1=0$$

Similarly, by deriving Equation (1) at the open-circuit point to obtain the expression of ${\left.\frac{dV}{dI}\right|}_{OC}$ and substituting it into Equation (7), Equation (11) can be expressed as

(11) $$\left(Rso-Rs\right)\left(\frac{1}{Rsh}+\frac{Is}{nVT}\exp\left(\frac{Voc}{nVT}\right)\right)-1=0$$

Above five equations, i.e., Equations (2), (3), (5), (10), and (11), are used to determine the analytical expressions of these five parameters. In order to get more accurate and efficient parameter values, two reasonable approximations need to be considered in these equations. The first one is *I_s_*exp(*V_oc_*/*nV_T_*) >> *I_s_*exp(*I_sc_R_s_*/*nV_T_*) due to *V_oc_* >> *I_sc_R_s_*, compared with the former, the term *I_s_*exp(*I_sc_R_s_*/*nV_T_*) can be ignored. In addition, when the value of the term *I_s_*exp(*I_sc_R_s_*/*nV_T_*) is too small and has little impact on the whole equation, it can also be replaced by 0. The second one is *R_sh_* >> *R_s_*. Thus, 1 + *R_s_*/*R_sh_* ≈ 1 and *R_sho_* ≈ *R_sh_* are also valid.

According to these two approximations, the expressions of five parameters can be extracted. However, the analytical equations for determining parameters generally cannot use too many approximate conditions. This may lead to low accuracy of parameters, which makes the calculation results unreliable and unsatisfactory. Therefore, it is necessary to reduce the use of approximation as much as possible and retain important conditions in the calculation. The detailed explanation is presented as follows.

Under the condition of constant irradiance, by taking Equation (2) into Equation (3) and eliminating *I_ph_*, and then using the first approximation *I_s_*exp(*V_oc_*/*nV_T_*) >> *I_s_*exp(*I_sc_R_s_*/*nV_T_*), Equation (12) can be obtained as

(12) $$Is\exp\left(\frac{Voc}{nVT}\right)=Isc\left(1+\frac{Rs}{Rsh}\right)-\frac{Voc}{Rsh}$$

In Equation (10), (*I_s_*/*nV_T_*)exp(*I_sc_R_s_*/*nV_T_*) is much smaller than the rest and after simplification, yielding:

(13) $$\frac{Rsho}{Rsh}=1+\frac{Rs}{Rsh}$$

According to Equation (13), Equation (12) can be rewritten as

(14) $$Is\exp\left(\frac{Voc}{nVT}\right)=\frac{IscRsho}{Rsh}-\frac{Voc}{Rsh}$$

Now, Equations (11), (13), and (14) need to be substituted into Equation (5). First, the *I_s_*exp(*V_oc_*/*nV_T_*) of Equation (5) needs to be replaced by the right part of Equation (14). Then, Equation (13) is used to replace *R_s_*/*R_sh_* in Equation (5). After replacing these parts above, an intermediate equation can be obtained as

(15) $$\frac{\left(Isc-Im\right)Rsho-Vm}{Rsh}=Is\exp\left(\frac{Vm+RsIm}{nVT}\right)$$

Both sides of the Equation (15) are represented by logarithmic computation, yielding:

(16) $$\ln\left[\left(Isc-Im\right)Rsho-Vm\right]-\ln Rsh=\ln Is+\frac{Vm+RsIm}{nVT}$$

Second, Equation (14) is expressed as *I_s_* on the left, the remaining part is on the right, and the right part is used to replace *I_s_* in Equation (16). Another intermediate equation can be obtained as

(17) $$\ln\left[\left(Isc-Im\right)Rsho-Vm\right]=\ln\left(IscRsho-Voc\right)-\frac{Voc}{nVT}+\frac{Vm+RsIm}{nVT}$$

Finally, Equation (11) needs to be rewritten as *R_s_* on the left and the remainder on the right, and then replace *R_s_* of Equation (17) with the remainder on the right, yielding:

(18) $$\ln\left[\left(Isc-Im\right)Rsho-Vm\right]-\ln\left(IscRsho-Voc\right)=\frac{ImRso+Vm-Voc}{nVT}-\frac{Im}{Isc\frac{Rsho}{Rsh}-\frac{Voc}{Rsh}+\frac{nVT}{Rsh}}$$

In the above part, we only use the first approximation instead of using the two approximations synchronously as the conventional method. This is mainly because *R_s_*/*R_sh_* is much bigger than *I_s_*exp(*I_sc_R_s_*/*nV_T_*) and the latter is more complex. This behavior effectively reduces the use of approximation conditions, which is very helpful to improve the accuracy of the parameters. However, in Equation (18), considering *R_sho_* ≈ *R_sh_* has very little effect on the whole equation, Equation (18) can be replaced by Equation (19) as follows.

(19) $$\ln\left[\left(Isc-Im\right)Rsho-Vm\right]-\ln\left(IscRsho-Voc\right)=\frac{ImRso+Vm-Voc}{nVT}-\frac{Im}{Isc-\frac{Voc}{Rsho}+\frac{nVT}{Rsho}}$$

It is worth noting that we need to solve a quadratic Equation (19) with one unknown parameter *n*. In order to avoid negative numbers and complex numbers, we choose the negative root as the solution of the parameter *n*, i.e.,

(20) $$n=\frac{-\sqrt{4ABCRsho+{\left(ACRsho+ImRsho-B\right)}^{2}}-\left(ACRsho+ImRsho-B\right)}{2A\cdot V_{T}}$$

Here *A*, *B*, and *C* are symbolled as $A=\ln\left[\left(Isc-Im\right)Rsho-Vm\right]-\ln\left(IscRsho-Voc\right)$,B=ImRso+Vm−Voc, $C=Isc-\frac{Voc}{Rsho}$.

According to the order of calculation and considering the second approximation, i.e., 1 + *R_s_*/*R_sh_* ≈ 1 and *R_sho_* ≈ *R_sh_* in Equation (12), *I_s_* can be extracted as

(21) $$Is=\left(Isc-\frac{Voc}{Rsho}\right)\exp\left(-\frac{Voc}{nVT}\right)$$

Similarly, using the approximation *R_sho_* ≈ *R_sh_* in Equation (11), *R_s_* can be extracted as

(22) $$Rs=Rso-\frac{1}{\frac{1}{Rsho}+\frac{Is}{nVT}\exp\left(\frac{Voc}{nVT}\right)}$$

According to Equation (11) and the above three parameters, i.e., *n*, *I_s_*, and *R_s_*, *R_sh_* can be extracted as

(23) $$Rsh=\frac{1}{\frac{1}{Rso-Rs}-\frac{Is}{nVT}\exp\left(\frac{Voc}{nVT}\right)}$$

Finally, by substituting Equations (20)–(23) into Equation (2) and the above four parameters, *I_ph_* can be extracted as

(24) $$Iph=Isc\left(1+\frac{Rs}{Rsh}\right)+Is\left(\exp\left(\frac{IscRs}{nVT}\right)-1\right)$$

Therefore, the five parameters of the single diode modeling for solar cells can be extracted from Equations (20)–(24) in sequence.

## 3. Verifications and Discussions

In this part, *I-V*, *P-V*, relative error, absolute error curves, and root-mean-square error (RMSE) are used to verify and compare the accuracy and effectiveness of the proposed parameter extraction strategy. In the verification process, the absolute error represents the absolute difference between the measured value and the real value, and the relative error is calculated by the ratio of the absolute error to the real value, and the result is expressed in the form of percentage. On the one hand, when we extract and compare parameters through a set of initial values, these characteristic curves can clearly show the experimental errors of different methods. In addition, RMSE can also evaluate the quality of all parameter extraction strategies. On the other hand, the performance of the proposed parameter extraction strategy in different cases should also be considered. These points are mainly reflected in PV technologies, irradiance, and temperature. Thus, in these cases, it is important and necessary to evaluate the fitting results of *I-V* and *P-V* curves between the experimental data and the results obtained by using the extracted parameters. Of course, the absolute error curves and RMSE are also obtained to better verify the performance of the method. The detailed verification results and discussion are as follows.

**Figure 3:**
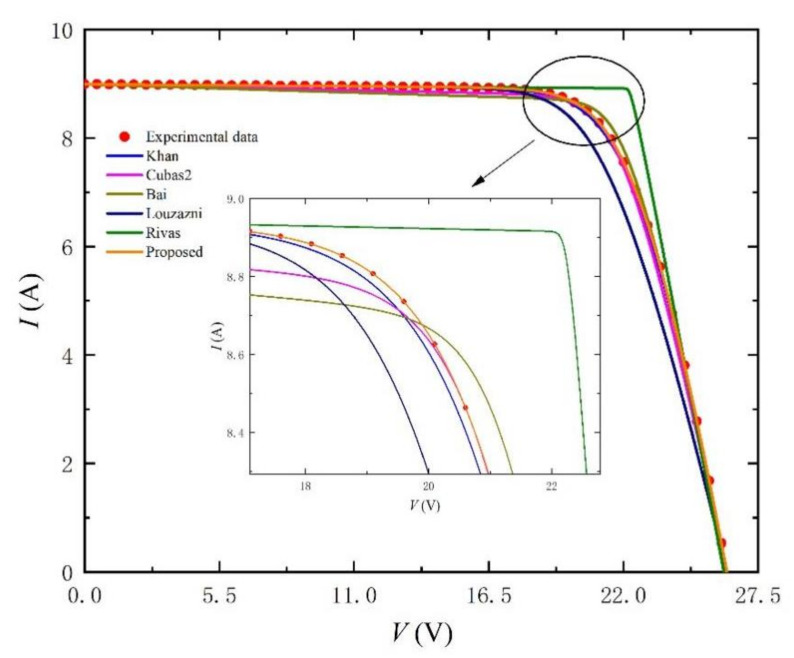
*I-V* curves simulated by using setting initial value and the extracted parameters listed in Table 1 at G = 1000 W/m^2^ and T = 25 °C.

**Figure 4:**
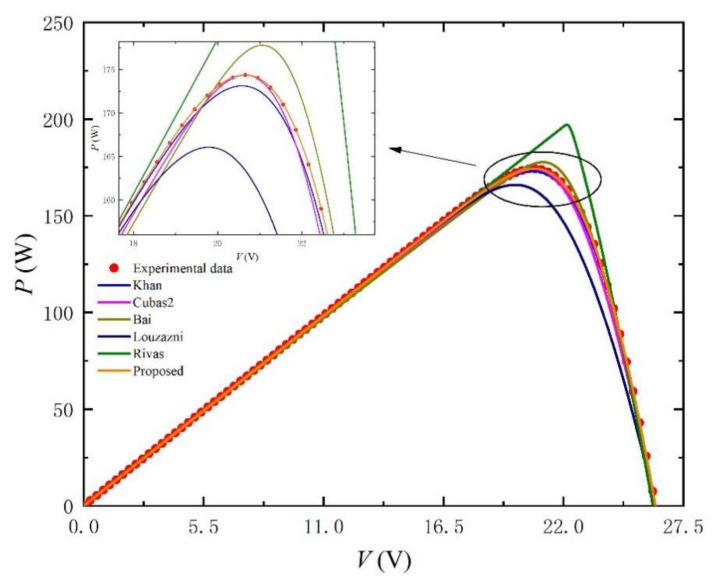
*P-V* curves simulated by using setting initial value and the extracted parameters listed in Table 1 at G = 1000 W/m^2^ and T = 25 °C.

According to the set of initial values, the simulation results are shown in Figure 3 and Figure 4. The comparison results of the parameters are shown in Table 1. First, we fix a set of initial values in Table 1 as reference (Setting). Second, after processing the reference data, we get the five key points mentioned in the second part. Finally, we use these key points to extract parameters so as to compare the proposed method with other methods in the previous literature and draw the corresponding *I-V*, *P-V* and absolute error percentage curves. We can observe from Figure 3 and Figure 4 that only our *I-V* and *P-V* curves agree to the experimental data (scatter points), which is significantly different from other methods. In particular, the part with large gap has been enlarged in Figure 3 and Figure 4 for better observation. All methods are simulated under 1000 W/m^2^ and 25 °C, and the obtained parameter results are shown in Table 1.

**Figure 5:**
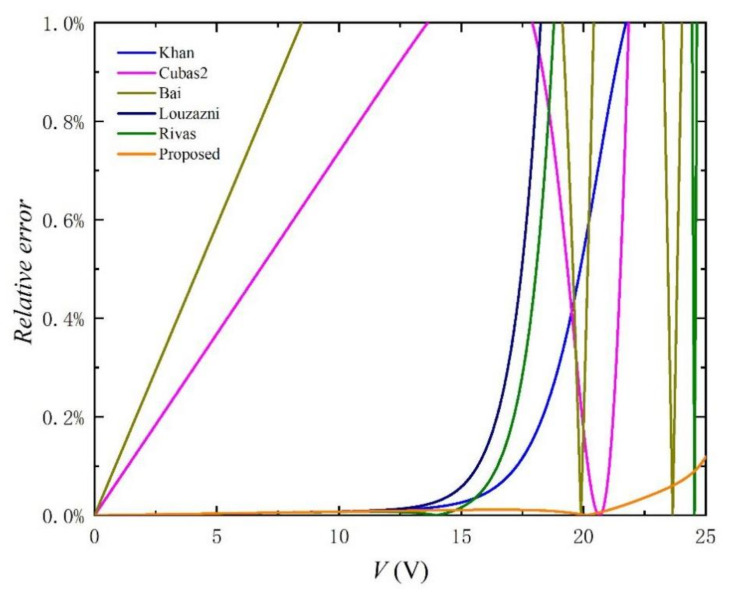
Relative error of *I-V* curves simulated by using setting initial value and the extracted parameters listed in Table 1.

It is obvious that the absolute error percentage curve shown in Figure 5 and the RMSE in Table 1 clearly reflecting that the error of the parameter extraction strategy proposed in this paper is the smallest, and the performance of this method is the best. It is worth noting that in Figure 5, it can be observed that the relative error of the proposed method remains almost below 0.1% within the effective range. Compared with other methods, the difference is obvious and the error is smaller, which fully meets the accuracy requirements of the simulations. In addition, the RMSE value of the proposed method in Table 1 is reduced by at least one order of magnitude, compared with other methods, which further reflects the advantages of this method.

**Figure 6:**
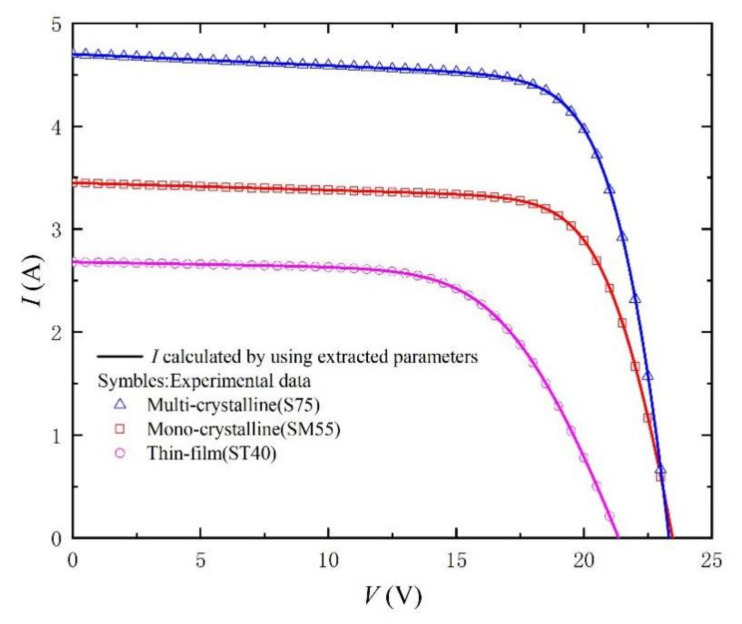
*I-V* curves measured from experimental data [33] for different PV modules and calculated by using the extracted parameters listed in Table 2.

**Figure 7:**
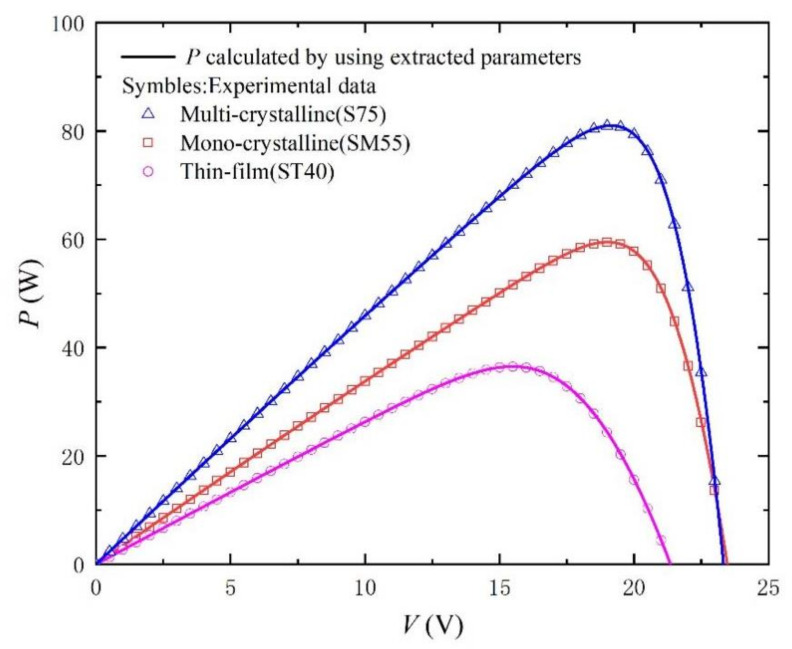
*P-V* curves measured from experimental data [33] for different PV modules and calculated by using the extracted parameters listed in Table 2.

**Figure 8:**
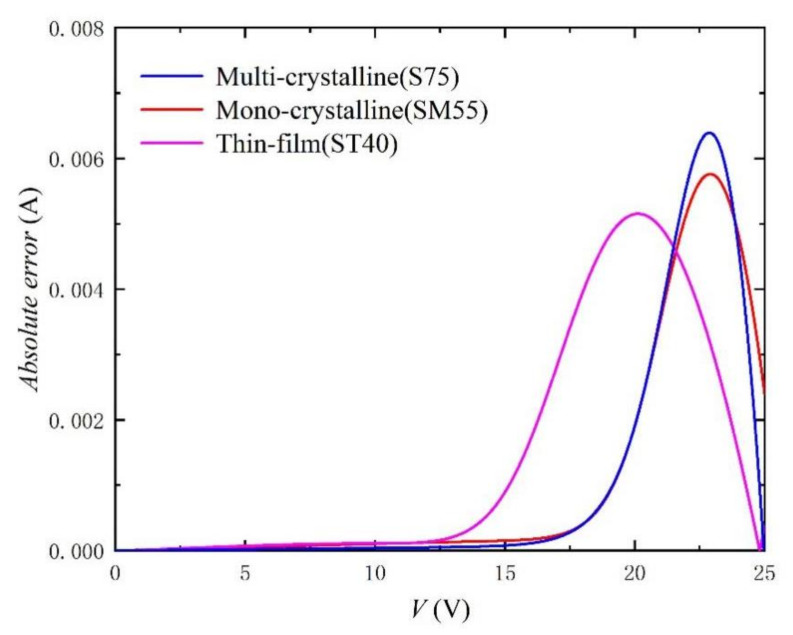
Absolute error curves between experimental data [33] and using the extracted parameters listed in Table 2.

In order to verify the practicability and effectiveness of this method, the parameter extraction results and calculated RMSE of three different photovoltaic modules (Mono-crystalline, Multi-crystalline, and Thin film) in the literature [33] are recorded in Table 2. Irradiance and temperature are still 1000 W/m^2^ and 25 °C, respectively. The corresponding *I-V* and *P-V* curves are shown in Figure 6 and Figure 7. We can still observe that the curve obtained by our proposed method is very consistent with the experimental data (scatter points) recorded in the literature. Figure 8 shows the absolute error curves of different photovoltaic technologies, and verifies the high precision and good practicability of our proposed method in photovoltaic technology. It should be noted that there are obvious peaks around V = V_OC_ in Figure 8. This is because in the process of curve fitting, the first approximation exists in the form of exponent. Due to the large range of abscissa and rapid change of exponent, the error will be accumulated and amplified, and there will be an obvious wave crest phenomenon. Of course, relative errors are still smaller than 0.2%.

**Figure 9:**
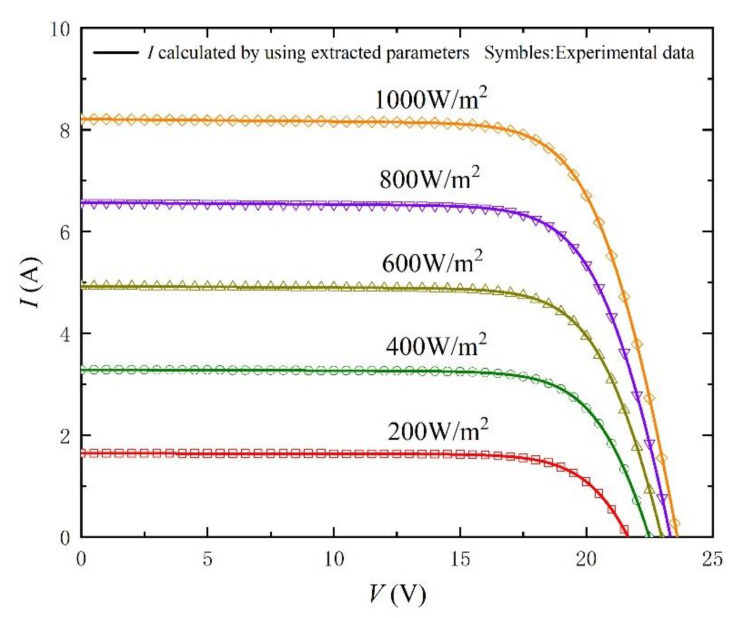
*I-V* curves measured from experimental data [33] at different irradiance levels and T = 25 °C, and calculated by using the extracted parameters listed in Table 3.

**Figure 10:**
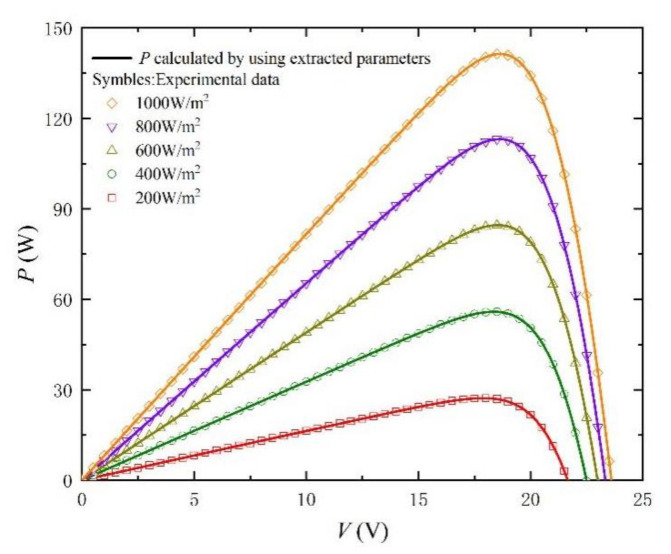
*P-V* curves measured from experimental data [33] at different irradiance levels and T = 25 °C, and calculated by using the extracted parameters listed in Table 3.

**Figure 11:**
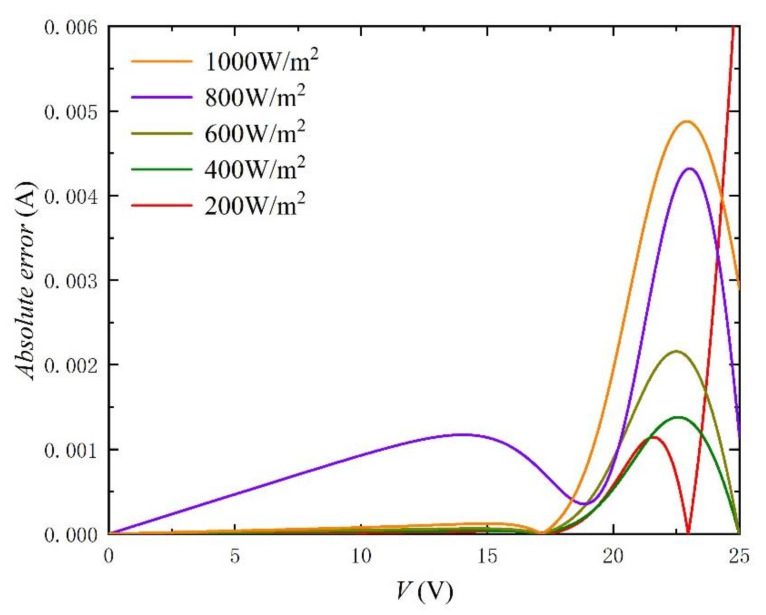
Absolute error curves between experimental data [33] and calculation results by using the extracted parameters listed in Table 3.

**Figure 12:**
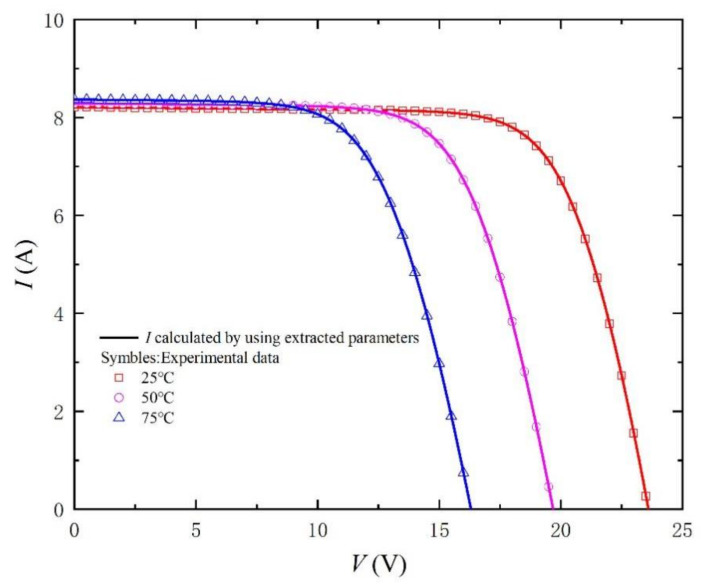
*I-V* curves measured from experimental data [33] at G = 1000 W/m^2^ and different temperatures, and calculated by using the extracted parameters listed in Table 4.

**Figure 13:**
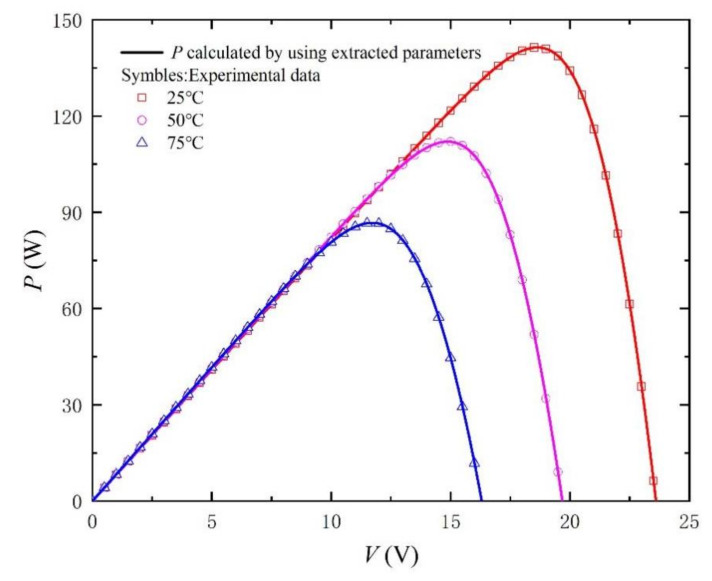
*P-V* curves measured from experimental data [33] at G = 1000 W/m^2^ and different temperatures, and calculated by using the extracted parameters listed in Table 4.

**Figure 14:**
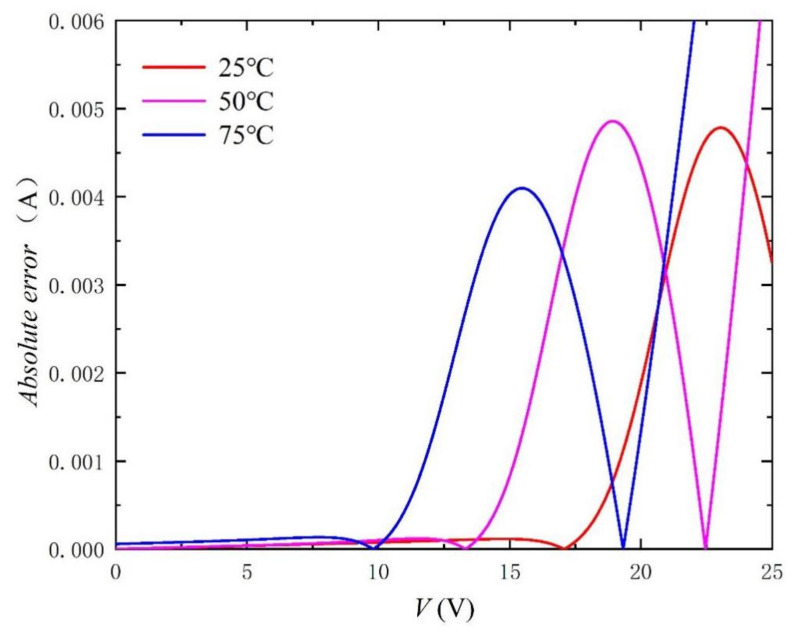
Absolute error curves between experimental data [33] and calculation results by using the extracted parameters listed in Table 4.

Similarly, in order to better verify the method, we carried out simulation experiments under different irradiances and temperatures. The comparison objects of the experiment are from the literature [33]. The extracted parameters and the calculated RMSE are recorded in Table 3 and Table 4. In particular, the values of RMSE are kept below 0.2%, which fully reflects the accuracy of the method. In addition, *I-V* and *P-V* curves with different irradiances and temperatures are shown in Figure 9, Figure 10, Figure 11, Figure 12, Figure 13 and Figure 14. It can be seen that the simulation results are consistent with the experimental data, which highlight the practicability of this method under different irradiances and temperatures. Finally, the absolute error curves of irradiance and temperature are shown in Figure 11 and Figure 14. In short, the above four experiments successfully verify the accuracy and practicability of the proposed parameter extraction strategy.

## 4. Conclusions

This paper presents an effective and accurate method to extract the model parameters of solar cells’ single diode model. First, the equivalent circuit and *I-V* curve of single diode model are given to obtain the required circuit equation and key points, including open circuit voltage, short circuit current, and maximum power point. Then, we get the other two constant conditions, *R_sho_* and *R_so_*, according to the slope of *I-V* curve at open-circuit point and short-circuit point. In order to overcome the problems of accuracy and complexity, we use the measures of approximations, retain important parts to obtain the simplified five equations, and then derive the five parameter expressions in order. Second, through setting initial values, the *I-V* and *P-V* characteristic curves are simulated, and the proposed method is compared with other different methods. According to the simulation results, our proposed method has the best applicability, which not only maintains good accuracy but also simplifies the parameter extraction process. Finally, the fitting and comparison are carried out by *I-V*, *P-V*, and absolute error curves under different PV technologies, irradiances, and temperatures. The obtained curves are in good agreement with the experimental data, which also proves the practicability of this method for different preparation conditions and environmental changes. In the future, it may be helpful to prepare solar cells in the face of changeable process conditions.

## Figures and Tables

**Table 1:** Parameter-extraction results and RMSE for *I-* and *P-V* Curves in Figure 3 and Figure 4 at G = 1000 W/m^2^ and T = 25 °C.

Methods (Year)	*n*	*I_s_* (A)	*R_s_* (Ω)	*R_sh_* (Ω)	*I_ph_* (A)	RMSE
Setting	40	1.0 × 10^−^^10^	0.3	300	9	-
Khan (2013)	42.5033	4.4532 × 10^−^^10^	0.2937	294.1	8.9999	0.0301
Cubas2 (2014)	28.0077	2.0138 × 10^−^^15^	0.3807	100.2	9.0251	0.1039
Bai (2014)	21.5822	4.4004 × 10^−^^20^	0.3807	71.5	9.0385	0.1132
Louzazni (2015)	39.9748	9.9279 × 10^−^^11^	0.4166	294.1	9.0037	0.3617
Rivas (2020)	1.7956	1.4471 × 10^−^^242^	0.4114	294.1	9.0035	0.3625
Proposed	39.9672	9.7823 × 10^−^^11^	0.2999	294.1	9.0001	0.0013

**Table 2:** Parameter-extraction results and RMSE for *I-* and *P-V* Curves in Figure 6 and Figure 7.

G (W/m^2^)	T (°C)	PV Modules	*n*	*I_s_* (A)	*R_s_* (Ω)	*R_sh_* (Ω)	*I_ph_* (A)	RMSE
1000	25	Multi-crystalline (S75)	45.0214	9.8695 × 10^−^^9^	0.1995	90.1	4.7103	0.0018
1000	25	Mono-crystalline (SM55)	42.3693	1.8463 × 10^−^^9^	0.4136	140.8	3.4601	0.0017
1000	25	Thin-film (ST40)	55.8424	1.0658 × 10^−^^6^	1.0651	232.6	2.6922	0.0021

**Table 3:** Parameter-extraction results and RMSE for *I-* and *P-V* Curves in Figure 9 and Figure 10.

T (°C)	G (W/m^2^)	*n*	*I_s_* (A)	*R_s_* (Ω)	*R_sh_* (Ω)	*I_ph_* (A)	RMSE
25	200	46.6459	2.8645 × 10^−^^8^	0.2978	1111.1	1.6435	0.0002
25	400	46.6232	2.8407 × 10^−^^8^	0.2652	555.6	3.2873	0.0003
25	600	46.6298	2.8482 × 10^−^^8^	0.2458	370.3	4.9309	0.0006
25	800	46.7473	2.9888 × 10^−^^8^	0.2315	285.7	6.5743	0.0013
25	1000	46.6454	2.8656 × 10^−^^8^	0.2212	222.2	8.2182	0.0016

**Table 4:** Parameter-extraction results and RMSE for *I-* and *P-V* Curves in Figure 12 and Figure 13.

G (W/m^2^)	T (°C)	*n*	*I_s_* (A)	*R_s_* (Ω)	*R_sh_* (Ω)	*I_ph_* (A)	RMSE
1000	25	46.6413	2.8607 × 10^−^^8^	0.2212	222.2	8.2182	0.0015
1000	50	43.3215	7.3686 × 10^−^^7^	0.2397	222.2	8.2977	0.0018
1000	75	40.4268	1.2025 × 10^−^^5^	0.2583	222.2	8.3771	0.0017

## Data Availability

The data presented in this study are available on a reasonable request from the corresponding author.
